# Half metal-to-metal transition and superior transport response with a very high Curie-temperature in CoFeRuSn: strain regulations†

**DOI:** 10.1039/d5ra01305d

**Published:** 2025-04-11

**Authors:** Farwa Rani, Bassem F. Felemban, Hafiz Tauqeer Ali, S. Nazir

**Affiliations:** a Department of Mechanical Engineering, College of Engineering, Taif University Kingdom of Saudi Arabia; b Department of Physics, University of Sargodha 40100 Sargodha Pakistan safdar.nazir@uos.edu.pk +92-334-9719060

## Abstract

Magnetic materials with a high Curie temperature (*T*_C_) and superior transport behavior are interesting prospects for spintronics and energy conversion devices. The present investigation deals with various traits of the CoFeRuSn quaternary Heusler alloy that were examined under strain (biaxial [110]/hydrostatic [111]) using first-principles calculations. The T1 phase is the ground state configuration relative to the T2 and T3 ones, which is thermodynamically stable owing to a negative formation enthalpy (Δ*H*_f_) and high cohesive energy (*E*_coh._). It is established that the material exhibits a half-metallic (HM) ferromagnetic (FM) phase holding an energy gap of 0.42 eV in the spin-minority channel. Moreover, Co/Fe/Ru has spin moment of 1.59/3.13/0.53 *μ*_B_ with electronic configuration of (3d7) t^3^_2g_↑t^3^_2g_↓e^1^_g_↑e^0^_g_↓/(3d^6^) t^3^_2g_↑t^3^_2g_↓e^2^_g_↑e^0^_g_↓/(4d^5^) t^3^_2g_↑t^2^_2g_↓e^0^_g_↑e^0^_g_↓ employing 
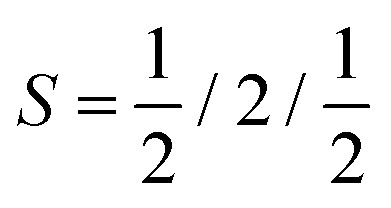
 which is further confirmed by spin-magnetization density isosurfaces. Exceptionally, a giant *T*_C_ of 779 K further enhances its potential for practical realization. Additionally, a large figure of merit (*ZT*) of 0.93 and n-type carriers indicates a good thermoelectric performance of the material. Surprisingly, a HM-to-metal transition occurs at −4% in both types of compressive strains. Furthermore, *ZT* reaches ∼1.0 under applied strains, whereas *T*_C_ increases by 11% for −5% strain. Hence, this study demonstrates that the CoFeRuSn motif contains multifunctional aspects, enhancing its potential for various applications in spintronics and thermoelectric energy harvesting.

## Abstract 2

Spin-magnetization density and energy band gap of the unstrained and strained (biaxial ([110]) and hydrostatic ([111])) CoFeRuSn quaternary Heusler alloy.

## Graphical abstract

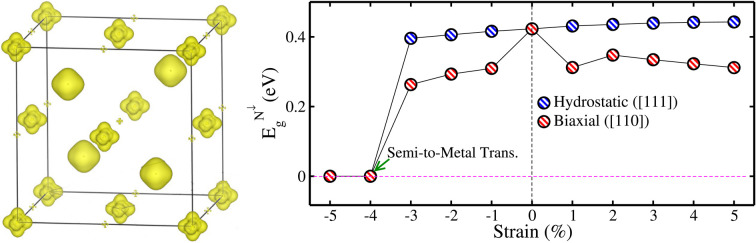

## 1 Introduction

Spintronics has emerged as a novel field since the 1990s,^1,2^ which incorporates the electron spin^3^ in contract to conventional electronics, where transport information is mostly dependent on their charge. In this way, fully spin-polarized (SP = 100%) called half-metallic (HM) ferromagnets (FM) are widely used in the development of high-performance spin-based devices,^4,5^ which have several advantages over traditional semiconductor (SC) electronic devices including being non-volatile,^6^ and having a low power consumption^7^ and high integration.^8^ Thus, higher electron polarizability is required and HM magnetic materials^9^ emerge as the ideal option for potential applications such as in spin diodes,^10^ spin valves,^11,12^ spin filters,^13,14^ memory chips,^15^ magnetic random access memories based on spin-transfer torque,^16^ magnetic tunnel junctions (MTJ),^17^ tunnel magnetoresistance (TMR), and giant magnetoresistance (MR).^18^ Recently, research has focused heavily on the unique electronic structure of the HM magnetic materials, where Heusler alloys (HAs) have gained special importance because of their interesting physical properties such as high *T*_C_ (Curie temperature (temp.)), tunable electronic structure, and a wide range of lattice constants making them ideal candidates for MTJs fabrication.^19^ In particular, the first experimental prediction of the HM feature in NiMnSb by de-Groot *et al.*, in 1983, sparked a lot of work in finding the HAs have unusual magnetism.^20^ Similarly, Cu_2_MnAl was also considered the first HM, which was identified in 1903 by German chemist Fredrich Heusler.^21^

Likewise, equiatomic quaternary HAs (QHAs) with 1 : 1 : 1 : 1 stoichiometry^22^ are the most recently identified class of HM materials and are a type of rare material with 4d or 5d transition metal (TM) elements with HM phases.^23^ It is found that they provide a wide range of materials with desired qualities: lower structure disorder, low power consumption, stable HM properties against strain, and better compositional and atomic arrangement flexibility as compared to full and half HAs.^24^ For instance, Özdoğan *et al.* used density functional theory (DFT) calculations to study 60 LiMgPdSn-type QHAs and discovered that the majority of them (40 alloys) exhibited HM behavior,^25^ where the remaining are SCs without a spin gap. In this fashion, many studies are being done on Co-based QHAs due to their outstanding potential for spintronics applications, reliable experimental synthesis, and structural stability.^26^ Block *et al.* first highlighted the possible use of Co_2_Cr_0.6_Fe_0.4_Al QHAs in spintronics by observing a significant negative magneto resistivity at room temp. when a minor external magnetic field was present.^27^ In the following year, Galanakis used DFT analysis to estimate the HM phase in numerous QHAs.^28^ For instance, CoFeTiSn and CoFeVGa are synthesized using the arc melting process.^29^ Additionally, the molecular beam epitaxy approach was successfully used to manufacture CoFeVSi.^30^ Some other QHAs which have been synthesized experimentally are CoRhMnGe,^31^ CoFeMnZ (Z = Al, Ga, Si, and Ge),^32^ CoRuMnGe and CoRuVZ (Z = Al, Ga),^33^ where results show that CoRhMnGe MTJ possesses an extremely large TMR value. CoFeMnZ (Z = Al, Ga, Si, and Ge) were identified as potential HM FMs with high *T*_C_ and CoRuMnGe/CoRuVAl/CoRuVGa were predicted to have a nearly HM FM state with high SP of 91/89/93%. Recently, an *ab initio* investigation has reported the existence of numerous Co-based QHAs with HM properties including CoFeCrP,^34^ CoFeTiAs,^35^ CoZrMnSb,^36^ CoFeTiZ (Z = Ge/Sb),^37^ CoFeTiSn,^22^ CoXZeGe (X = Rh/Ru),^38^ CoMRhSi (M = Cr/Mn),^39^ CoX′ZrGa (X′ = V, Cr),^40^ CoZrTiX (X = Ga, Si, and Sn),^41^ and CoNbTiX (X = Al, Ga, and In),^42^ which are considered to be ideal for utilization in the spintronic field.

Additionally, it is possible to precisely manipulate the electronic structure by strain regulation, which affects how electrons behave within a material and makes it possible to develop materials with particular electronic and transport traits that are interrelated. Recent advances in the research into QHAs using DFT techniques have revealed vital insights into their many physical traits, highlighting their potential applications in spintronics and energy-related technologies.^43,44^ These studies have thoroughly explored the impact of elemental substitutions on HM state, stability, and thermoelectric (TE) efficiency, providing techniques for improving material performance.^45,46^ Additionally, they also emphasize the importance of disorder, strain, and electronic correlations in designing functional qualities, providing a better knowledge of material design concepts. Notably, high *T*_C_ HAs are ideal for spintronic and TE applications because they maintain stable magnetic behavior even at high temp., making them ideal for high-temp. devices like magnetic sensors and energy harvesters.^47^ Strain can improve several physical aspects such as thermal conductivity (*k*), electrical conductivity (*σ*), optical properties, and mechanical strength. Recent materials science research on strain modulation has provided important new insights into the manipulation of phase transitions and electronic characteristics in a variety of compounds, which has shown a notable improvement in the material aspects under strain. Wang *et al.*^48^ studied in detail the effect of uniform strain and tetragonal distortions on the HM properties of the QHAs RuMnCrZ (Z = P/As/Sb) and observed that the HM state in RuMnCrSb remains in the range of −6 to +5%. Along with this, they also thoroughly studied the impact of uniform strain on the HM properties of QHAs FeMnCrZ (Z = P, As, Sb, Bi, Se, and Te)^49^ and reported that a uniform lattice expansion can induce a ferrimagnetic (FiM) to FM phase transition in FeMnCrZ (X = As and Te) and the effects of the tetragonal distortions turn out to be negligible. Ray *et al.*^50^ investigated the magnetostructural and strain-induced electronic structure effects on ZrRhTiZ (Z = Al and In), where they showed that the HM FM behavior is retained within −2% to +2% strain in contrast with the anomalous spin flip at −1% and +1% strains. Similarly, Yan *et al.*^51^ discovered that the QHA TiZrCoIn could retain its HM nature under the hydrostatic (hydro.) strain ranging from −10% to +7.6% and tetragonal strain ranging from −19% to +27%. Jia *et al.*^52^ demonstrated that FeMnZnZ (Z = Si, Ge, Sn, and Pb) could maintain its HM behavior with uniform strain between −13% and +14%.

Consequently, it is important to investigate how long the HM FM state lasts in these materials when subjected to strain. The material’s intrinsic features are modified by perturbing its structure such as by applying external strain. The lattice mismatch between the SC substrate and epitaxial thin films causes in-plane compressive (comp.) or tensile (tens.) strain perpendicular to the thin-film growth, resulting in tetragonal distortions. In this light, we have selected a recently proposed QHA CoFeRuSn (CFRS)^53^ to examine how strains (biaxial (biax.)) ([110]) and hydro. ([111])) affect the system’s electronic structure. At first, we determined the energetically stable phase of CFRS by taking into account three nonequivalent structural configurations. After that the electronic and magnetic traits of the stable atomic configuration of the unstrained (unstr.) CFRS are investigated by calculating the total/partial density of states (TDOS/PDOS) as well as band structures and total/partial spin magnetic moment (*m*_t_/*m*_s_) along with three-dimensional (3D) spin-magnetization isosurfaces. Next, the TE performance of the system is analyzed in terms of various parameters. Eventually, *T*_C_ is computed using the Heisenberg Hamiltonian model.

## 2 Computational and structural details

In this work, DFT calculations were carried out using the self-consistent full-potential linearized-augmented plane-wave (FP-LAPW) approach as implemented in the WIEN2k code.^54^ The exchange–correlation functional is utilized in terms of the generalized gradient approximation (GGA)^55^ within the Wu–Cohen (WC) scheme. Also, on-site Coulomb interactions (Hubbard parameter (*U*)) are considered to account for the strong correlation effects on the 3/5d TM orbitals with values of 5.0, 4.6, and 2.4 eV on the Co, Fe, and Ru ions, respectively.^56^ Basically, *U* is utilized to correct the energy-eigenvalues of the TM d-orbitals as described in ref. 56. In addition, we used the modified Becke–Johnson (mBJ) potential to get the correct electronic structure of the system. The plane-wave cutoff is set to *R*_mt_ × *K*_max_ = 7 with *G*_max_ = 24 and a maximum value of *l*_max_ = 12 is selected for the wave function expansion inside the atomic spheres. To establish good self-consistency, a grid of 72 *k*-points in the irreducible wedge of the Brillouin zone was taken from a 12 × 12 × 12 *k*-mesh. Moreover, structure self-consistency is adopted for a total charge/energy (*E*_t_) convergence of 10^−6^ C/10^−6^ Ry.

**Fig. 1:**
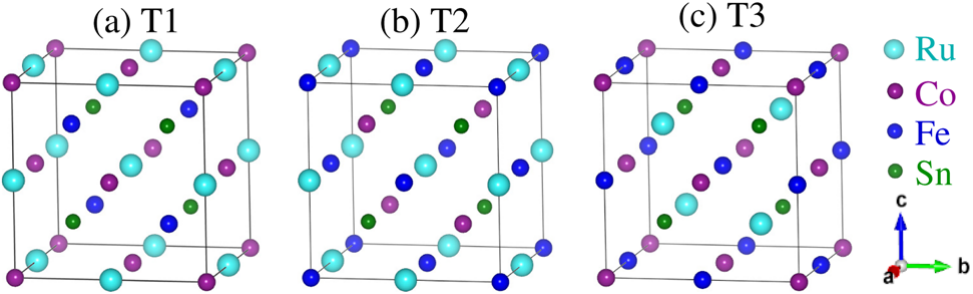
Crystal structures of the CoFeRuSn quaternary Heusler alloy in three different configurations: (a) T1, (b) T2, and (c) T3.

The CFRS QHA is a LiMgPdSn-type compound that has four interpenetrating face-centered cubic sublattices with a space group of *F*4̄3*m* (No. 216).^57^ The typical structure of QHA is denoted as XX′YZ and within the primitive cell, the four atoms are situated at the Wyckoff positions of 4a(0, 0, 0), 4c(1/4, 1/4, 1/4), 4b(1/2, 1/2, 1/2), and 4d(3/4, 3/4, 3/4). Here, X, X′, and Y are the d-TM elements and they lie at 4a, 4c, and 4b sites; Z is from s–p block elements (main group element) and it stays at the 4d site. If the position of Z is fixed at 4d, then different arrangements of X, X′, and Y-elements at 4a, 4c, and 4b Wyckoff positions generally result in three different types of structures such as type1 (T1), type2 (T2), and type3 (T3) as shown in Fig. 1(a)–(c), respectively.^58^ For the T1 structure, the Wyckoff positions of 4a(0, 0, 0)/4c(1/4, 1/4, 1/4)/4b(1/2, 1/2, 1/2)/4d(3/4, 3/4, 3/4) were occupied by the Co/Fe/Ru/Sn atoms. Similarly, the T2 structure can be constructed by placing the Co/Fe/Ru/Sn atoms at the Wyckoff positions of 4c(1/4, 1/4, 1/4)/4a(0, 0, 0)/4b(1/2, 1/2, 1/2)/4d(3/4, 3/4, 3/4). Similarly, the T3 structure can be created by placing Co/Fe/Ru/Sn ions at 4a(0, 0, 0)/4b(1/2, 1/2, 1/2)/4c(1/4, 1/4, 1/4)/4d(3/4, 3/4, 3/4) (also listed in Table 1).

**Table 1:** Atomic configurations of the CoFeRuSn quaternary Heusler alloy with three unique arrangements of Co, Fe, Ru, and Sn atoms at different Wyckoff positions

Structure	4a (0, 0, 0)	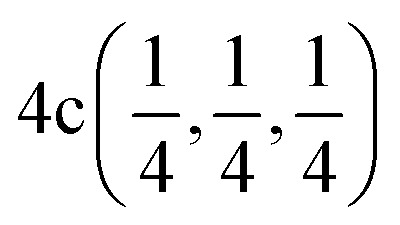	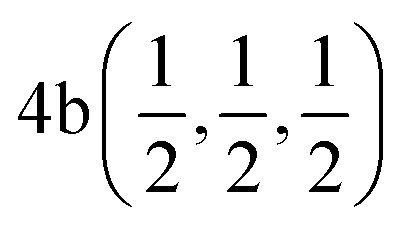	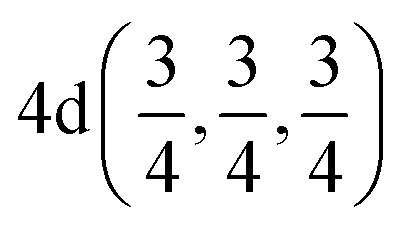
Type I	Co	Fe	Ru	Sn
Type II	Fe	Co	Ru	Sn
Type III	Co	Ru	Fe	Sn

## 3 Results and discussions

### 3.1 Unstrained system

**Fig. 2:**
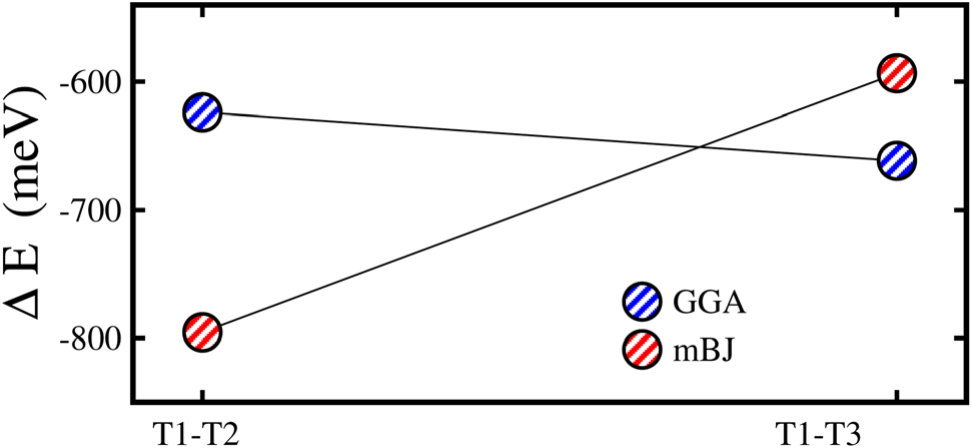
Calculated total energy difference (Δ*E*) between the type1 (T1) and type2 (T1–T2)/type3 (T1–T3) configurations of the CoFeRuSn quaternary Heusler alloy.

First, the stable phase of the system is analyzed by computing the *E*_t_ of all three configurations and it is found that the T1 phase has the lowest *E*_t_ compared to that of the T2/T3 within GGA and mBJ-GGA methods showing a significant energy gap (*E*_g_) from the T2 and T3 phases (see Fig. 2). Therefore, the T1 configuration is adopted while calculating the physical properties of the system. Now, we investigated the structural stability of the CFRS in its stable T1 phase by calculating the formation enthalpy (Δ*H*_f_) as:1
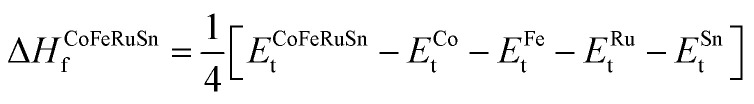
Here *E*^CoRuSn^_t_ refers to the ground state *E*_t_ of the primitive cell, whereas *E*^Co^_t_, *E*^Fe^_t_, *E*^Ru^_t_, and *E*^Sn^_t_ are the ground states *E*_t_ of the Co, Fe, Ru, and Sn ions within the respective stable crystal/magnetic phases, correspondingly. The determined Δ*H*_f_ is −1.9 eV, where the “–” sign indicates that a material is chemically integrable and thermodynamically stable. Moreover, the cohesive energy (*E*_coh._), which determines the stability of interatomic bonds in a solid and provides additional information about the structural stability is computed as:2
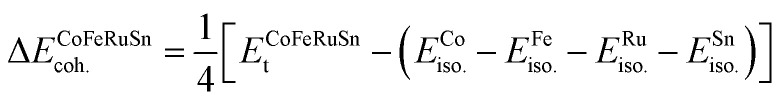
where *E*^CoRuSn^_t_ is the equilibrium *E*_t_ in the T1 stable phase for the CFRS and *E*^Co^_iso._/*E*^Fe^_iso._/*E*^Ru^_iso._/*E*^Sn^_iso._ is the isolated atomic energy of the Co/Fe/Ru/Sn element. Our results predict that *E*_coh._ is 4.41 (eV/atom), where its value indicates that once CFRS is synthesized, its chemical bonds can be maintained and will not disintegrate into free atoms, ensuring the structural stability of the system. Additionally, to ensure the dynamic stability of the motif, phonon dispersion curves are plotted in Fig. 1S of the ESI.† Each phonon band lies above zero (*i.e.*, this means that they have positive values), which ensure its dynamic stability as well.

**Fig. 3:**
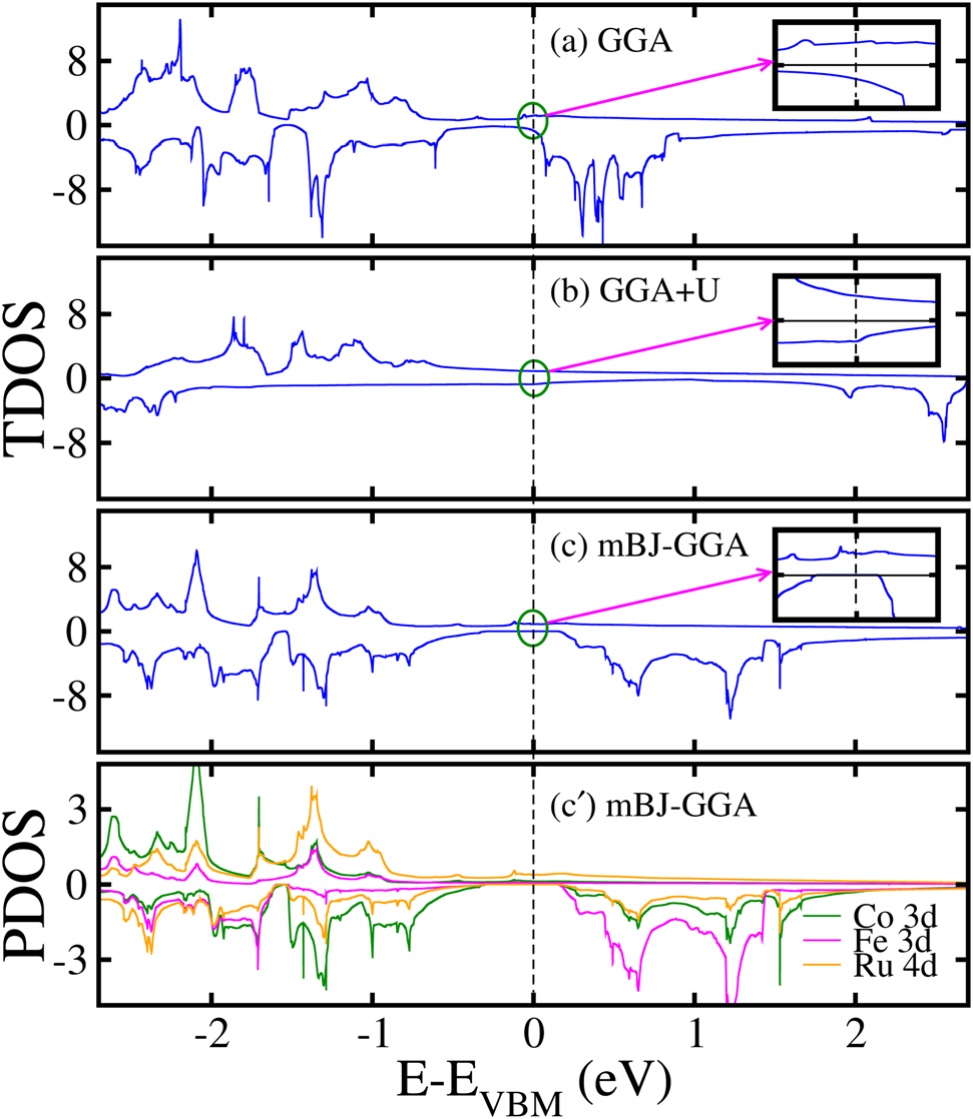
Computed (a/b/c) GGA/GGA + *U*/mBJ-GGA spin non-degenerate total density of states (TDOS) in states per eV and (c′) mBJ-GGA orbital-resolved partial density of states (PDOS) in states/eV on the Co 3d/Fe 3d/Ru 4d states in a stable T1 configuration for the unstrained CoFeRuSn quaternary Heusler alloy.

To investigate the electronic properties of the motif, we plotted the computed non-degenerate TDOS for the unstr. CFRS system within GGA/GGA + *U*/mBJ-GGA method in Fig. 3(a)–(c). From Fig. 3(a), one can see that within GGA method, the TDOS in the spin-majority/spin-minority channels (*N*↑/*N*↓) are non-degenerate and have few states crossing the Fermi level (*E*_F_) from valence band (VB) to conduction band (CB) in both channels (see the inset of TDOS for clarity), which verifies the metallic behavior of the unstr. system. Moreover, it is found that this metallic behavior is maintained even when the GGA + *U* approach is used as depicted in Fig. 3(b), indicating that the metallic character of the material is not much changed by the addition of on-site Coulomb interactions (see the inset for TDOS, which plots the region very near *E*_F_ for clarity). Surprisingly, substantial changes occur in the electronic structure when the mBJ-GGA technique is used as *N*↑ stays metallic (*i.e.*, few states appear at *E*_F_). In contrast, the *N*↓ becomes an insulator owing to an *E*_g_ of 0.42 eV, leading the system into HM FM state (see Fig. 3(c)), which is consistent with the earlier theoretical and experimental research.^53,59,60^ However, here we would like to mention that one can get the more accurate results with a more powerful functional called HSE06, which is implemented in the VASP code. The is because the mBJ-GGA method has its own limitation due to a complex entanglement between electron exchange and correlation terms. Moreover, this HM gap is a bit smaller as compared to the other HM systems at room temp.^61,62^ However, it will prevent the spin-flip transition for HM at room temp. value at least because its *T*_C_ value is very high. To gain a thorough knowledge of the states around the *E*_F_, we plotted the non-degenerate PDOS on the Co 3d, Fe 3d, and Ru 4d orbitals in Fig. 3(c′) from the mBJ-GGA scheme. It is observed that d-states of the TM ions are responsible for the metallic nature of the alloy in the *N*↑ and show a definite *E*_g_ of 0.42 eV in the *N*↓, giving it a SC nature. The splitting of d-states and the formation of the *E*_g_ are mostly caused by d–d hybridizations. It has been observed that the Ru-4d orbitals predominantly contribute to the TDOS near *E*_F_ with a minor contribution from the Co/Fe 3d orbitals. Hence, it is also worth noting that Ru 4d states play a pivotal role in generating the *E*_g_ in the *N*↓ between VB and CB (see Fig. 3(c′)). Moreover, spin–orbit coupling (SOC) effects are taken into account due to the heavy Ru element and the TDOS plotted in Fig. 2S(a) and 2S(b) of the ESI† within the GGA-mBJ + SOC and GGA + *U* + SOC methods, respectively. It is found that system becomes metal with the incorporation of SOC effects, which was expected.

**Fig. 4:**
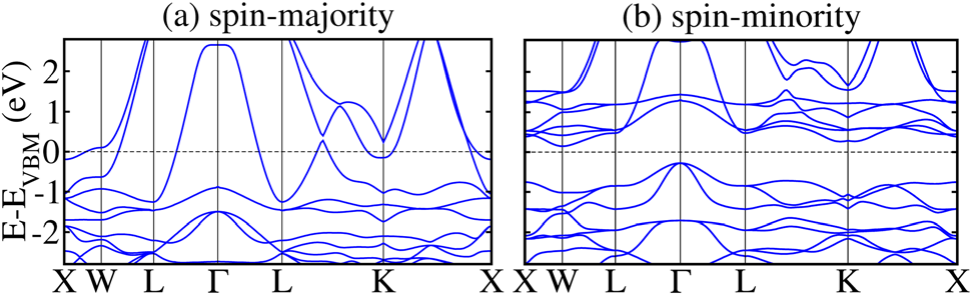
mBJ-GGA calculated spin non-degenerate band structures for the spin-majority/spin-minority channel (a/b) in of the CoFeRuSn quaternary Heusler alloy in a stable T1 configuration.

The electronic state of the system is further verified by calculating the non-degenerate band structures. This provides a clear perspective of the TDOS behavior in an unstr. motif using the GGA/GGA + *U* and mBJ-GGA method as shown in Fig. 3S/4S of the ESI† and Fig. 4, respectively. It is found that the band structure calculated using the GGA/GGA + *U* technique (see Fig. 3S/4S of the ESI†) validates the computed TDOS in Fig. 3(a)/3(b) and further confirms the system conductivity. It turns out to be metallic as the VB and CB curves touch the *E*_F_ in both channels. In the case of mBJ-GGA, few bands develop at the *E*_F_ in the *N*↑ and ensure the channel metallicity (see Fig. 4(a)). Contrary to that SC behavior is observed in the *N*↓ keeping an *E*_g_ of 0.42 eV as no band crosses the *E*_F_ (see Fig. 4(b)), which further assists the TDOS in Fig. 3(c). As CFRS HA has an *E*_g_ of 0.42 eV in the *N*↓, which is small in comparison to other half-HAs such as XIVNiSn, which has an *E*_g_ of around 0.5 eV according to DFT simulations, where most are p-type having a wider *E*_g_ ranging from 0.5 to 1 eV.^63^ This may be limit its applications, however, our strain calculations show that it maintains its HM state up to a reasonable range of −3% to +5% strains (discussed later).

**Fig. 5:**
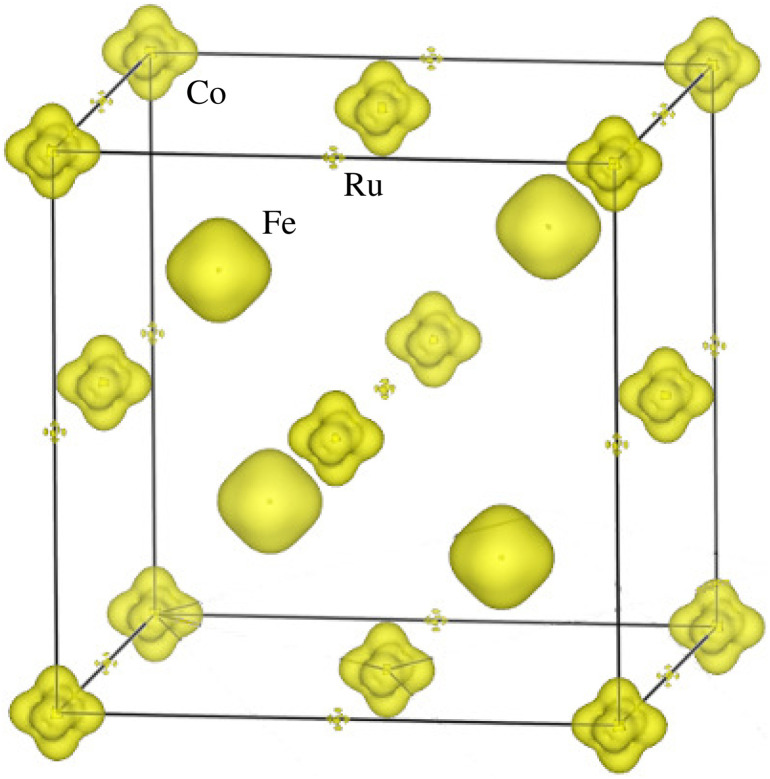
Computed mBJ-GGA spin-magnetization density isosurfaces with an iso-value of ±0.05 e Å^−1^ for the CoFeRuSn quaternary Heusler in a stable T1 configuration.

Next, we discuss the magnetism in the CFRS structure by describing the *m*_t_/*m*_s_ of the magnetic ions. The calculated *m*_t_ of the motif is 5.00*μ*_B_ per f.u., which is consistent with the experimentally and theoretically observed value of 5.00*μ*_B_ per f.u.^53,60^ For a compound to be considered a HM FM, the estimated *m*_t_ must be an integer number according to the Slater–Pauling rule *M*_tot._ = *Z*_tot._ – 24, where *M*_tot._ and *Z*_tot._ denote the *m*_t_ per f.u. and total number of valence electrons, respectively. Indeed, we observed that *m*_t_ determined using the mBJ-GGA technique is an integer value (5.00*μ*_B_), which satisfies the Slater–Pauling rule, further confirming that CFRS is a HM. The calculated *m*_s_ on the Co/Fe/Ru ion is 1.59/3.13/0.53*μ*_B_ along with a very small value of 0.0*μ*_B_ observed on the Sn ion as well. Similarly, the determined *m*_s_ on the Co/Fe/Ru/Sn ions are 1.32/3.04/0.58/0.02 and 1.81/3.99/0.37/0.11*μ*_B_ within the GGA and GGA + *U* methods, respectively. The discrepancy in *μ*_B_ magnitude can be easily understood as in the mBJ-GGA and GGA + *U* methods extra energy is provided to the systems which reduces the hybridization among ions and results in larger *m*_s_ values than those of the GGA ones. Next, we computed and illustrated the 3D spin-magnetization density isosurfaces holding an isovalue of ±0.05 e Å^−1^ for the unstr. CFRS system (see Fig. 5) to better understand the induced *m*_s_ on the magnetic ions. One can see that Fe and Co ions have higher densities than Ru, indicating that they are the key contributors to *m*_t_. This supports the estimated *m*_s_ for these ions as well. Furthermore, Co is in the +2(3d^7^) state holding 
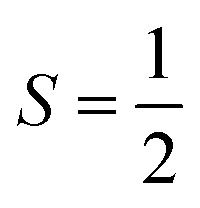
 with an electron distribution of t^3^_2g_↑t^3^_2g_↓e^1^_g_↑e^0^_g_↓. It is clear that t_2g_ orbitals are entirely occupied in the *N*↑ and *N*↓, whereas e_g_ states are partially filled in *N*↑ and predominantly unoccupied in the *N*↓. Thus, only e_g_ orbital features are present in the isosurface of the Co ion (see Fig. 5). Equivalently, the Fe ion lies in a +2(3d^6^) state holding electron distributions of t^3^_2g_↑t^3^_2g_↓e^2^_g_↑e^0^_g_↓ with *S* = 2. Therefore, t_2g_ orbitals in the *N*↑/*N*↓ are fully/partially occupied, while e_g_ states are completely filled in the *N*↑ but remain unoccupied in the *N*↓. So, the combined influence of the t_2g_ and e_g_ states are visible in the isosurface densities of the Fe ion (see Fig. 5). Similarly, the spin-magnetization density isosurfaces of the Ru ion show that only t_2g_ orbital aspects are present as it is in a +3(4d^5^) state keeping electronic configurations of t^3^_2g_↑t^2^_2g_↓e^0^_g_↑e^0^_g_↓ employing 
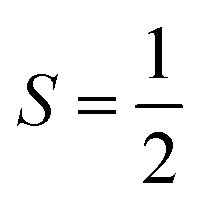
. This indicates that t_2g_ states are fully/partially occupied in the *N*↑/*N*↓ and e_g_ states are unoccupied in both channels. Therefore, the calculated Co/Fe/Ru *m*_s_ of 1.59/3.13/0.53 *μ*_B_ suggests that it remains in a +2(3d^7^)/+2(3d^6^)/+3(4d^5^) state.

Certainly, *T*_C_ is a significant factor in determining the feasibility of materials for spintronic applications since it indicates the stability of magnetic ordering at high temp. In this work, the *T*_C_ of CFRS was determined using the exchange coupling constants in terms of interatomic separations within the Heisenberg model, where the spin Hamiltonian is provided as:^64^3
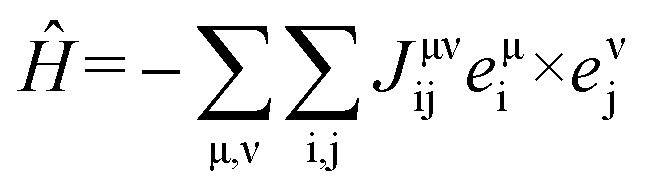
here *μ*/*ν* indicates distinct sub-lattices, while *i*/*j* denotes atomic position. The term *e*^μ^_i_/ *e*^ν^_i_ represents the alignment of the unit vector with *m*_s_ observed at site *i*/*j* in sub-lattice *μ*/*ν*. According to the Liechtenstein *et al.*^65^ approach, *J*^μν^_ij_ is calculated from the energy differences for the infinitesimally small variations in the orientations of a pair of spins. Therefore, after computing *J*^μν^_ij_, the *T*_C_ was estimated using the mean-field approximation as:^64^4
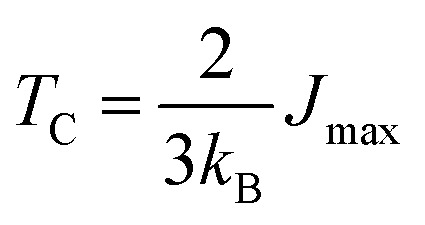
where *J*_max_ is the maximum eigenvalue of the *J*_eff._ matrix. The effective exchange coupling constant is given as 
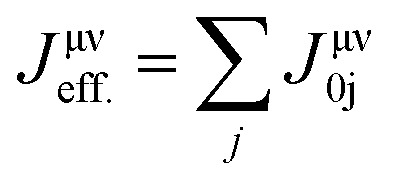
, where 0 is fixed inside the *μ* sub-lattice and *j* represents all atomic positions in the *ν* sub-lattice. Thus, the computed *T*_C_ of the CFRS motif is 779 K, indicating its high thermal stability and strong magnetic ordering. As CFRS exhibits a very high *T*_C_ along with a probable HM state, it is an attractive material for spintronic applications in magnetic storage technologies such as hard disk drives and magnetic random-access memory.^66^ Moreover, traditional HAs have drawbacks such as structural instability from off-stoichiometry and disorder, leading to poor mechanical properties.^67,68^ In contrast, the CFRS alloy is more stable due to its quaternary composition and reduced DO_3_ disorder. Furthermore, conventional HAs have low *T*_C_, making them unsuitable for high temp. applications.^39,69^ However, the present material has a very high *T*_C_, which makes it suitable for room temp. applications. Along with this, electronic characteristics present additional challenges as typical HAs often deviate from HM behavior due to defects and impurities.^68^ In contrast, CFRS maintains a HM phase even in the presence of defects.^53^

Next, we will investigate the TE aspects of the CFRS alloy to assess its efficiency in energy conversion applications. TE materials are critical for turning waste heat into electrical energy, providing a sustainable solution to the global energy dilemma. HM FM material with its distinctive electronic structures and great charge carrier mobility, holds considerable promise for TE applications. In this work, we investigate important transport parameters including the Seebeck coefficient (*S*), *σ* per unit relaxation time 
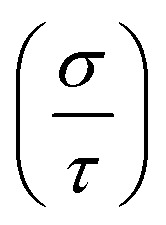
, power factor 
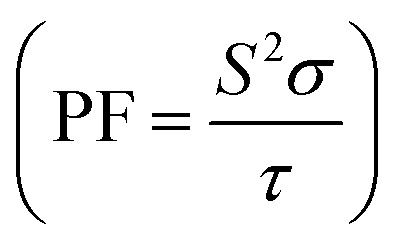
, electronic *κ*
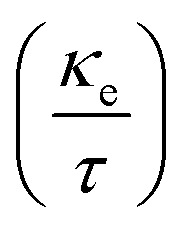
, and figure of merit 
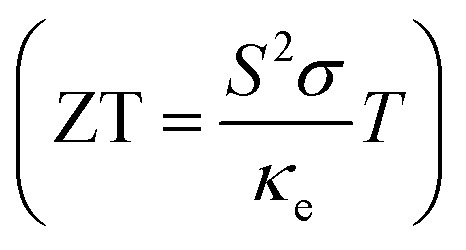
 using the BoltzTraP code under the constant relaxation time (*τ*) of 1 × 10^−14^ s. The *τ*, which governs the charge carrier dynamics, has a significant impact on the TE performance. The BoltzTraP1 code does not directly compute *τ*, but our results show that it follows a metallic trend for the *σ*↑ and a semiconductor-like behavior for the *σ*↓, indicating distinct charge carrier scattering processes. The phonon mean free path (*λ*) is important in determining the lattice thermal conductivity (*κ*_ph_). Phonon scattering in HM and semiconducting materials, such as CFRS, is dominated by interactions with charge carriers and defects, which can result in higher TE efficiency at certain temp. The observed decrease in *ZT*↓ at higher temp. may be ascribed to greater phonon–phonon scattering, which decreases *λ* and boosts *κ*_ph_, therefore reducing *ZT*.^70^

**Fig. 6:**
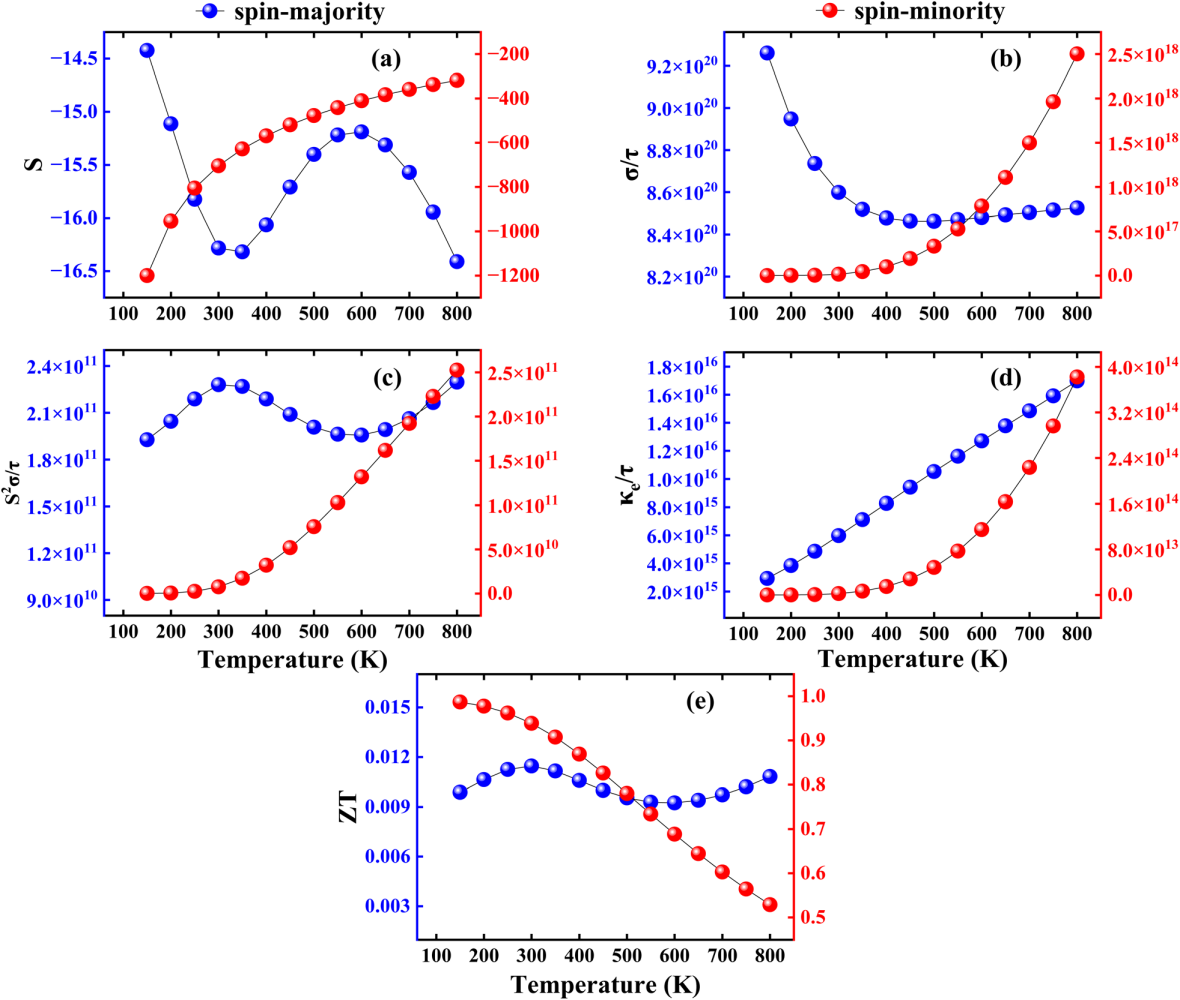
Computed (a) Seebeck coefficient (*S*) in μV K^−1^, (b) electrical conductivity per relaxation time 
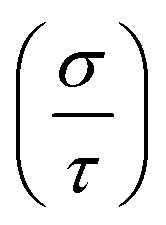
 in Ω^−1^ m^−1^, (c) power factor per relaxation time 
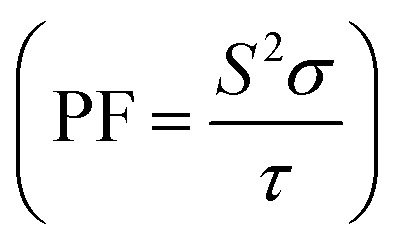
 in W m^−1^ K^−2^ s^−1^, (d) electronic thermal conductivity per relaxation time 
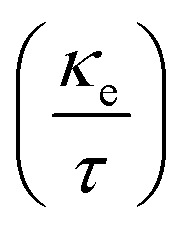
 in W m^−1^ K^−1^ s^−1^, and (e) figure of merit (*ZT*) as a function of temperature for the spin-majority (blue color)/spin-minority(red color) channel in the CoFeRuSn quaternary Heusler alloy.

The *S* defines a material’s capacity to create electromotive force (EMF) from an applied temp. gradient through the material or in other words, the efficiency of the thermocouple. The electronic movement generates thermo EMF, resulting in a voltage in microvolts per Kelvin (μV K^−1^). The *S* for CFRS spin-majority (*S*↑) and spin-minority (*S*↓) channels are negative over the temp. range of 150 K to 800 K, indicating n-type charge carrier dominance as depicted in Fig. 6(a). From 150 to 300 K, *S*↑ decreases somewhat from −14.4 to −16.2 μV K^−1^, whereas *S*↓ increases sharply from −1200.2 to −703.9 μV K^−1^ (becoming less negative), showing a considerable drop in TE voltage for the *S*↓. At 800 K, *S*↑ declines to −16.4 μV per K, while *S*↓ stabilizes at −317.7 μV K^−1^, indicating a slower rate of change with increasing temp. The temp. dependent evolution of the electrical conductivity 
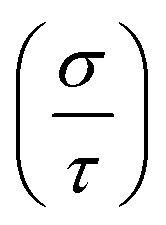
 for the spin-majority 
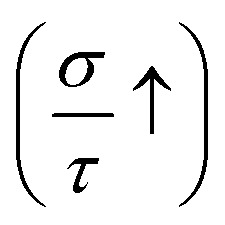
 and spin-minority 
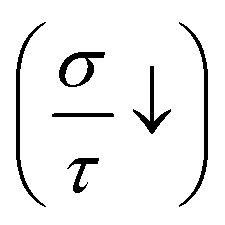
 channels is depicted in Fig. 6(b). At 150 K, 
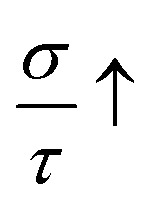
 drops from 0.92 × 10^21^ Ω^−1^ m^−1^ to 0.85 × 10^21^ Ω^−1^ m^−1^ at 300 K, indicating a metallic behavior. Beyond 300 K, 
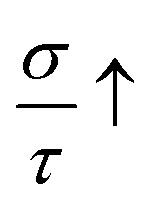
 is reasonably steady, reaching 0.85 × 10^21^ Ω^−1^ m^−1^ at 800 K.

The 
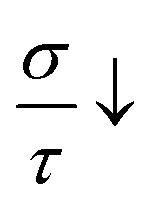
 has SC-like properties with 
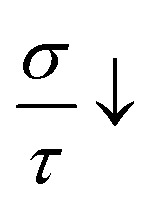
 growing considerably from 0.17 × 10^14^ Ω^−1^ m^−1^ at 150 K to 0.25 × 10^19^ Ω^−1^ m^−1^ at 800 K. This significant rise in conductivity is due to spin-minority states having a lower DOS at the *E*_F_, resulting in initially lower conductivity than the 
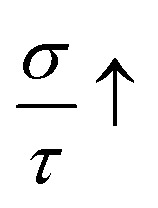
. Our work shows a decrease in 
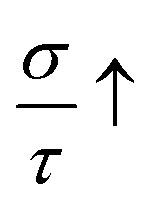
 and an increase in 
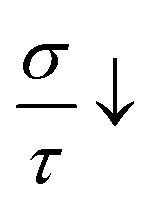
 in the CFRS, which is consistent with the theoretical study. Likely, the term PF determines whether a material is suitable for TE applications. Fig. 6(c) shows the PF for both spin-majority (PF↑) and spin-minority (PF↓) channels. At 300 K, PF↑ reaches 2.2 × 10^11^ W m^−1^ K^−2^ s^−1^, but PF↓ is substantially lower at 7.5 × 10^9^ W m^−1^ K^−2^ s^−1^. As the temp. rises to 800 K, PF↑ increases to 2.3 × 10^11^ W m^−1^ K^−2^ s^−1^, but PF↓ shows a large increase to 2.5 × 10^11^ W m^−1^ K^−2^ s^−1^, surpassing the spin-majority contribution. This pattern indicates that TE performance in the PF↓ increases considerably at increasing temp. emphasizing increased power production potential in this regime. The 
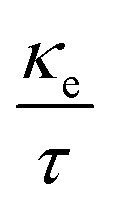
 of the CFRS shows a temp. dependency for both spin channels as illustrated in Fig. 6(d). As the temp. rises from 150 to 800 K, both channels exhibit an increasing trend, with 
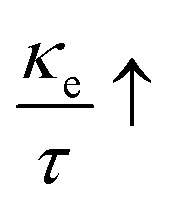
 reaching 1.7 × 10^16^ W m^−1^ K^−1^ s^−1^ and 
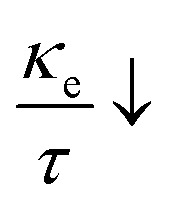
 rising to 3.8 × 10^14^ W m^−1^ K^−1^ s^−1^, showing improved electronic heat transport at higher temp. Finally, the ultimate performance of a material for TE applications is determined by *ZT*. The *ZT*↑ has a small value of 0.01 at 300 K, whereas the *ZT*↓ has a substantially larger value of 0.98/0.93 at 150/300 K as shown in Fig. 6(e). *ZT* is strongly influenced by electron and phonon transport, which determines the TE performance of the systems. Due to lower *κ*_e_ in the CFRS and greater *S*, the *N*↑ has a higher TE efficiency with *ZT*↓ reaching at 0.9869 at 150 K. On the other hand, *N*↓ exhibits a more metallic character, resulting in lower *ZT*↑ and greater *κ*_e_. Moreover, phonon transport is important at low temp., which usually suppresses the lattice thermal conductivity (*κ*_ph_) and increases *ZT*↓, but at higher temp., enhanced phonon–phonon scattering causes a decrease of *ZT*↓.^70^ These results highlight the interaction between phonon scattering and spin-dependent charge transport, hence enhancing the TE potential of CFRS. It is important to mention here that our predicted *ZT* of 0.93 in the CFRS HA is much greater than that of many recorded in the HM HAs having values ranging from 0.1 to 0.7.^71,72^ This suggests that CFRS has improved electronic and thermal transport capabilities, making it an attractive choice for TE applications.

First, we evaluated the mechanical stability of QHA CFRS and then used the elastic tensors (*C*_ij_) to figure out the thermal conductivity (*κ*_L_). The three independent elastic stiffness tensors are presented in Table 2, and meet the essential mechanical stability requirements and Born criteria: (*C*_11_ − *C*_12_) > 0, (*C*_11_ + 2*C*_12_) > 0, *C*_11_ > 0, and *C*_44_ > 0,^73^ confirming the material’s stability. The bulk modulus (*B*) quantifies a material’s resistance to deformation under pressure, while a higher shear modulus (*G*) indicates greater stiffness and resistance to shear stress, and a lower *G* suggests increased flexibility or ductility, with the Young’s modulus (*Y*) measuring the material’s stiffness under tension or compression. Furthermore, the *C*_11_/*C*_12_/*C*_44_ parameters are utilized to calculate the *B*, *G*, and *Y*, yielding values of 267.53/182.56/102.73 GPa, respectively, for the QHA CFRS system. Additionally, Pugh’s ratio (*B*/*G*)/Cauchy’s pressure (*C*_P_)/Poisson’s ratio (*ν*) were computed to determine the material’s ductile or brittle nature as summarized in Table 2. A material is considered brittle if B/G < 1.75, *ν* < 0.25, and *C*_P_ < 0, whereas if B/G, *ν*, and *C*_P_ are greater than 1.75, 0.25, and 0, respectively, the system will be ductile.^74^ The computed values of *B*/*G*, *C*_P_, and *ν* confirm the ductile nature of the material (see Table 2), which is in good agreement with the previously reported results by Gupta *et al.*^60^

**Table 2:** Computed elastic constants (*C*_11_/*C*_12_/*C*_44_), *B*/*G*/*Y* in GPa, Pugh’s ratio (*B*/*G*), Poisson’s ratio (*ν*), Cauchy’s pressure (*C*_P_) in GPa, density (*ρ*) in g cm^−3^, longitudinal/transverse/average sound velocity (*v*_l_/*v*_t_/*v*_avg._) in ms^−1^, and Grüneisen parameter (*γ*) for the CoFeRuSn quaternary Heusler alloy

*C*_11_	*C*_12_	*C*_44_	*B*	*G*	*Y*	*B*/*G*	*ν*	*C*_P_	*ρ*	*v*_t_	*v*_l_	*v*_avg._	*γ*
267.53	182.56	102.73	210.88	72.09	194.15	2.93	0.35	79.83	9.74	2720.6	5614.2	3057.3	2.13

**Fig. 7:**
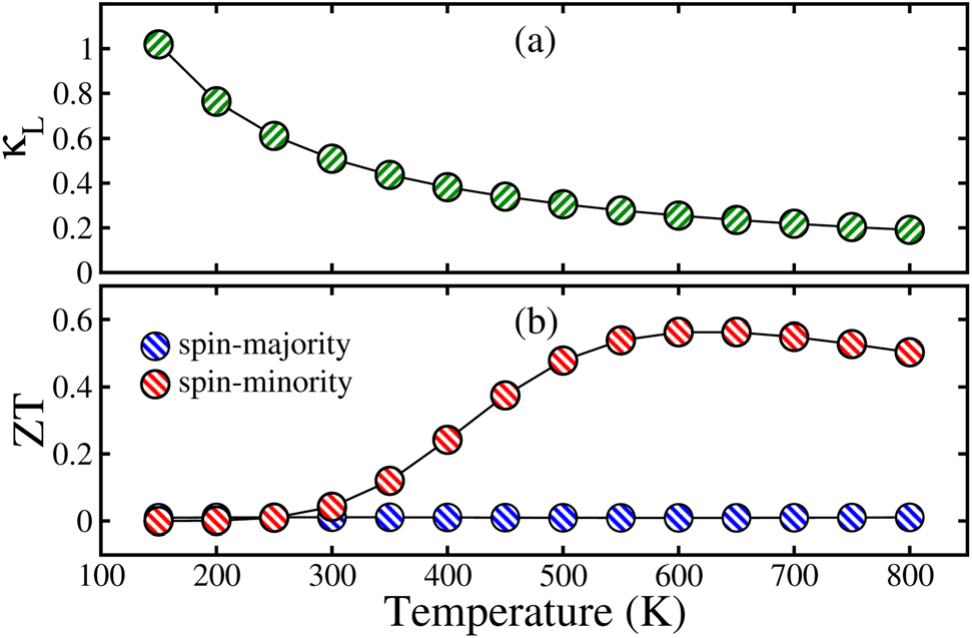
Variations of the (a) lattice thermal conductivity (*κ*_L_) in W mK^−1^, (b) figure of merit (*ZT*) for the CoFeRuSn over the temperature range of 150 to 800 K.

Now, we presented the computed *κ*_L_ in Fig. 7(a), by utilizing Slack’s model.^75^ The estimated *κ*_L_ exhibits a decreasing trend with increasing temp. due to growing anharmonic effects. Specifically, *κ*_L_ decreases gradually from 1.09 W mK^−1^ at 150 K to 0.19 W mK^−1^ at 800 K, as shown in Fig. 7(a). However, the room temp. (300 K) value of *κ*_L_ is 0.51 W mK^−1^. This temp. dependent reduction in *κ*_L_ underscores the potential of the material for use in TE devices, where low *κ*_L_ is crucial for enhancing performance. Finally, we present the *ZT* incorporating *κ*_L_ in Fig. 7(b). *ZT*↑ displays a constant behavior with temp. reaching 0.01 at 800 K due to its HM nature. Interestingly, *ZT*↓ shows a steady rise as a function of temp., peaking at 0.56 at 600 K (see Fig. 7(b)). Furthermore, at room temp., the *ZT*↑/*ZT*↓ value at ambient conditions is 0.01/0.04, which reveals that the overall *κ* is predominantly governed by the *κ*_L_, which substantially effects the *ZT* value.

### 3.2 Strained system

**Fig. 8:**
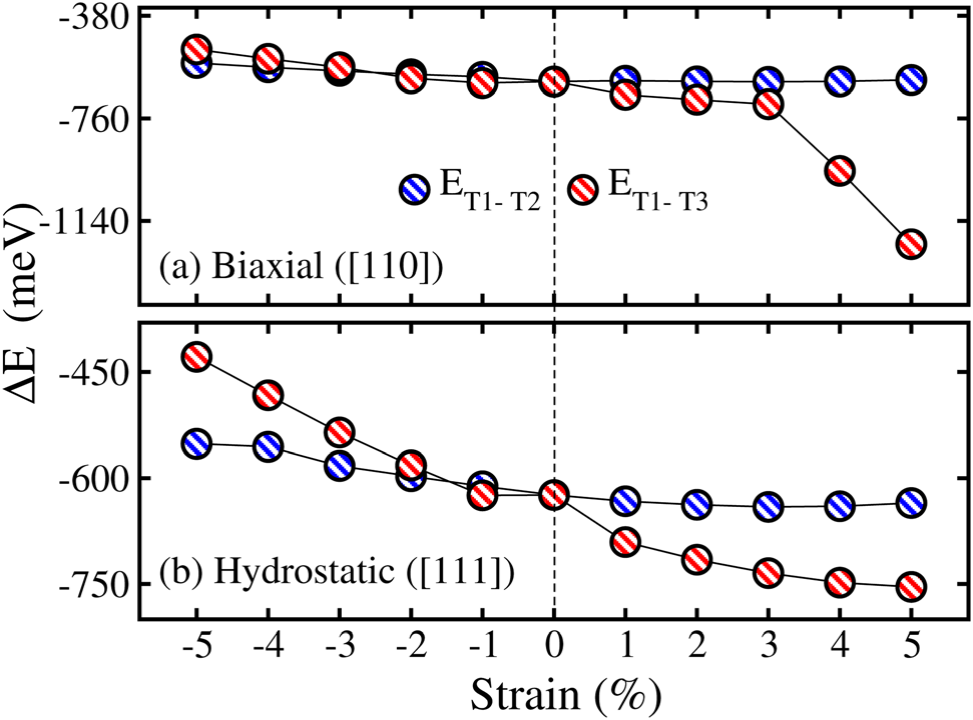
mBJ-GGA calculated Δ*E*_T1−T2_ (total energy difference between T1 and T2 phases)/Δ*E*_T1−T3_ (total energy difference between T1 and T3 phases) as a function of ±5% (a) biaxial ([110]) and (b) hydrostatic ([111]) strains in the CoFeRuSn quaternary Heusler alloy.

Here, we investigated the influence of biax. and hydro. strains on the physical aspects of the CFRS QHA by contracting and expanding the lattice constants up to ±5% relative to the equilibrium lattice parameter (0% value) along the [110] and [111]-directions, respectively. First, we investigated the structural durability of the system by calculating Δ*E* for the three structural configurations (T1, T2, and T3) under applied strains as presented in Fig. 8. One can see that T1 is the most stable configuration throughout the whole strain range owing to its lower energy than that of the T2 and T3 phases. It is clear that under comp. strains, the energy differences (Δ*E*_T1–T2_ and Δ*E*_T1–T3_) are moving towards zero, which means that these structures become less stable as compared to the 0% one. Conversely, tens. strained structures reveal more stability than that of the 0% as they move towards lower energies (become more negative). However, in both cases, Δ*E* remains negative, which verifies the robustness of the T1 phase in both types of strains. Consequently, the T1 phase is taken into consideration for further investigations. Afterward, to verify the thermodynamic stability at each strain value, the calculated Δ*H*_f_ under both strains is demonstrated in Fig. 5S(a) of the ESI.† It can be seen that Δ*H*_f_ is negative, indicating that all the strained systems are stable and can easily be grown in experiment under ambient conditions. It is demonstrated that when comp. strain is applied from −1% to −5%, the system becomes less stable due to the reduced negative amplitude of Δ*H*_f_ as compared to the 0% one. In contrast Δ*H*_f_ becomes more negative for tens. (+1% to +5%) strain structures than that of the 0%, signifying the greater strength (see Fig. 5S(a) of the ESI).† Moreover, Fig. 5S(b) of the ESI† explores computed *E*_coh._ values under strains, which remain positive at all strain levels, further verifying the material’s structural integrity. The system stability is somewhat diminished under comp. strain (−1% to −5%) as demonstrated by a lower amplitude of *E*_coh._ than that for 0%. However, tens. strains improve the material strength, resulting in a more negative *E*_coh._ values in comparison to 0% (see Fig. 5S(b) of the ESI).†

**Fig. 9:**
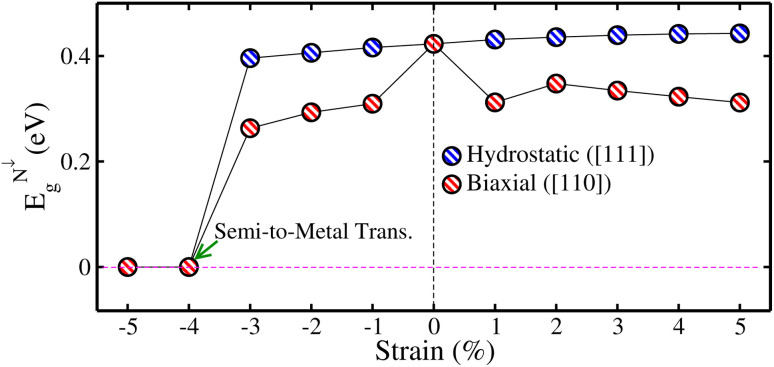
mBJ-GGA calculated energy gap in the spin-minority channel (*E*^*N*↓^_g_) as a function of ±5% biaxial ([110])/hydrostatic ([111]) strains in the CoFeRuSn quaternary Heusler alloy.

**Fig. 10:**
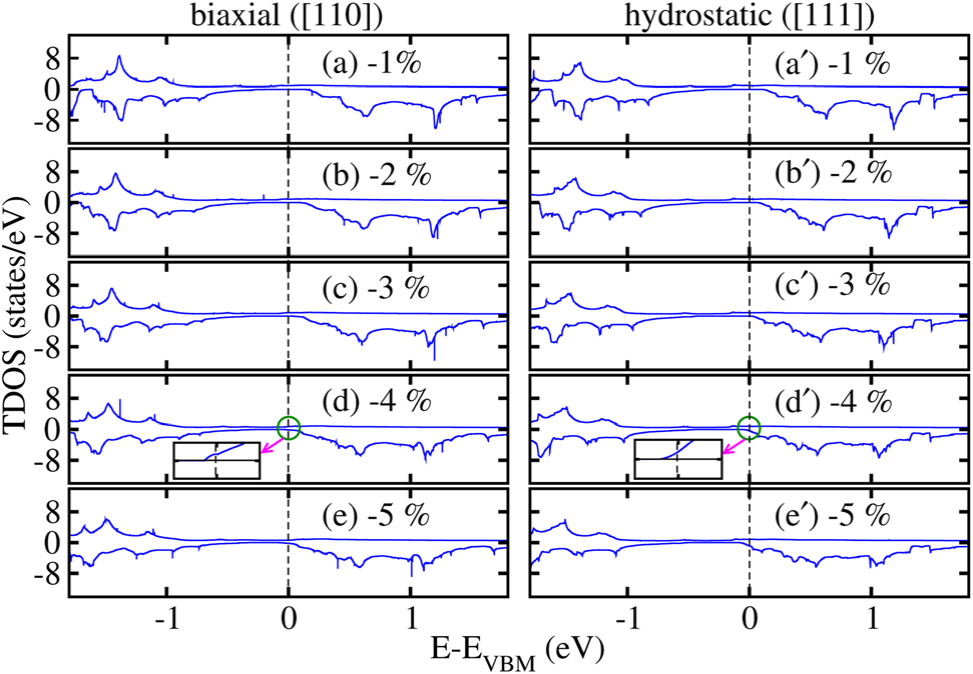
mBJ-GGA calculated spin-polarized total density of states (TDOS) for (a/a′) −1%, (b/b′) −2%, (c/c′) −3%, (d/d′) −4% and (e/e′) −5% biaxial ([110])/hydrostatic ([111]) compressive strains in the CoFeRuSn quaternary Heusler alloy.

Now, to investigate the strain-induced changes in the electronic structure of the CFRS motif, we plotted the computed *E*_g_ in the non-metallic channel (*E*^*N*↓^_g_) against strains in Fig. 9 because *N*↑ remains metallic for the whole strain range. For increasing hydro. tens. strain levels, *E*^*N*↓^_g_ gradually increases, demonstrating an improvement of the SC state in said channel. Conversely, *E*^*N*↓^_g_ decreases steadily under hydro. comp. strains and eventually becomes zero at −4%, displaying a transformation in electronic properties from SC to metal in the *N*↓. Likewise, *E*^*N*↓^_g_ becomes zero at −4% (see Fig. 9) under biax. comp. strain. Moreover, *E*^*N*↓^_g_ gradually decreases as tens. strain increases. For hydro. comp. strain, *E*_g_ decreases from 0.42 eV at 0% to 0.39 eV at −3%. The tendency of gradual increase in *E*_g_ as a function of tens. hydro. strain from 0.42 eV at 0% to 0.44 eV at +5% can be seen, demonstrating an improved insulating state under tens. strain. However, under biax. strain, *E*_g_ steadily declines from 0.42 eV at 0% strain to 0.26 eV at −3% and finally to 0.31 eV at +5%, indicating a reduction in the *E*_g_ with both comp. and tens. strains. This pattern suggests a strain-induced semiconductor-to-metal transition in the comp. regime, but tens. strain stabilizes the *E*_g_. Therefore, a transition from SC to a metallic state is evident at a critical comp. biax. strain of −4% in the *N*↓. Moreover, *E*^*N*↓^_g_ gradually decreases as tens. strain increases. Hence, it is predicted that a whole system exhibits a transition from HM to metal (as both channels become conductors) at a critical biax./hydro. −4% comp. strain. Qualitatively, the TDOS for the CFRS system under comp. strains ranging from −1% to −5% are plotted in Fig. 10. Our findings demonstrated that the system maintains its HM character up to ≤−3% biax./hydro. comp. strain as no substantial changes occur in the electronic structure (see Fig. 10(a)–(c)/(a′)–(c′)). However, at −4% comp. strain (see Fig. 10(d)/(d′)), the TDOS in the *N*↓ shifts towards lower energies and crosses the *E*_F_, resulting in a metallicity (see the inset of TDOS for clarity, where a few states appear at *E*_F_ in the *N*↓). Thus, this confirms the electronic phase transformation from HM-to-metal. A similar transition is also obtained for −5% biax./hydro. comp. strain as displayed in Fig. 10(e)/(e′).

**Fig. 11:**
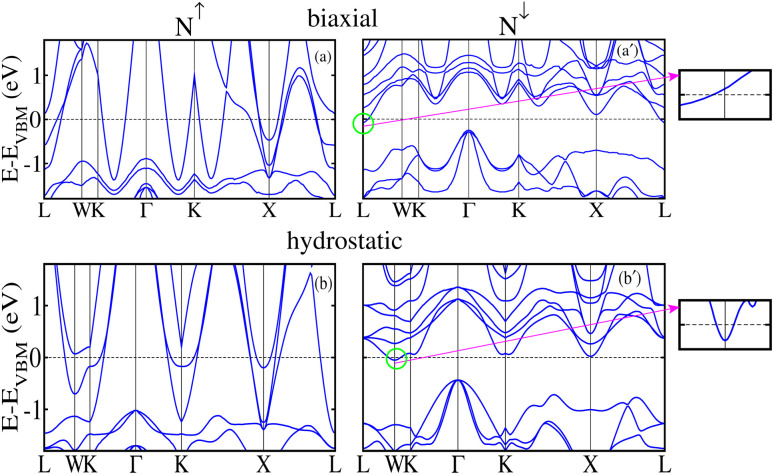
mBJ-GGA calculated spin-polarized spin-majority/spin-minority (*N*↑/*N*↓) band structure for a critical −4% (a)/(a′) biaxial ([110]) and (b)/(b′) hydrostatic ([111]) compressive strain in the CoFeRuSn quaternary Heusler alloy.

**Fig. 12:**
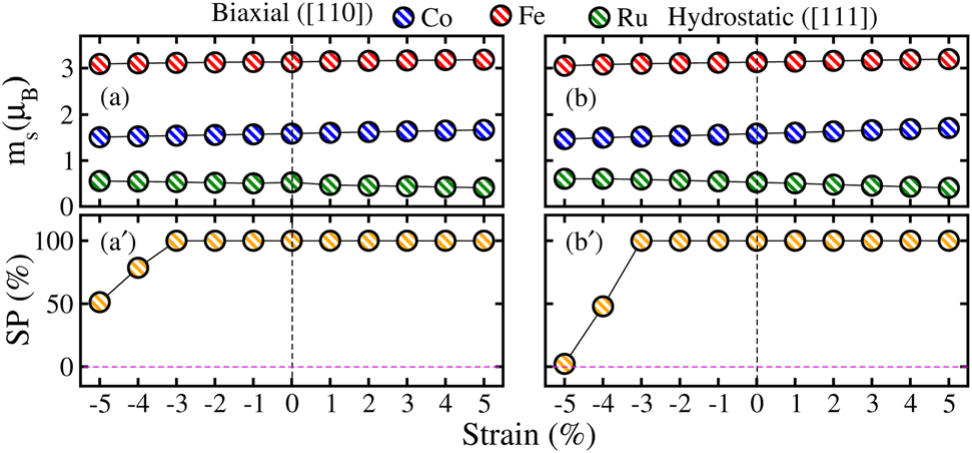
mBJ-GGA calculated partial spin magnetic moments on the Co/Fe/Ru ion as well as spin polarization (SP) for ±5% (a)/(a′) biaxial ([110]) and (b)/(b′) hydrostatic ([111]) strains in the CoFeRuSn quaternary Heusler alloy.

To further understand the electronic transitions in strained motifs, the mBJ-GGA computed SP band structures under critical −4% biax. and hydro. comp. strains are presented in Fig. 11. As few bands overlap at *E*_F_ in the *N*↑, this confirms the metallic behavior for biax. strain (see Fig. 11(a)). Likewise, a few bands in the *N*↓ (see Fig. 11(a′) pass through the *E*_F_ from CB to VB indicating the said channel is metallic. For clarity, an enlarged portion of the *N*↓ band structure further verifies the metallic state, where few bands are crossing the *E*_F_. Similarly, the plotted SP bands in Fig. 11(a′)/(b′) at a −4% critical hydro. comp. strain provides the same information as both channels are metallic. Hence, this provides additional evidence that a transition occurs from HM to metal at a crucial −4% biax./hydro. comp. strain as the TDOS does in Fig. 10(d)/(d′). After that, we examined the effect of strain on the magnetic traits of the CFRS system by calculating *m*_s_ on the Co, Fe, and Ru ions under biax./hydro. strain in Fig. 12(a)/(b). The results reveal that *m*_s_ on the Co/Fe ion exhibits a gradual increase from 1.60 to 1.67 *μ*_B_/3.15 to 3.18 *μ*_B_ under biax. tens. strains from +1% to +5%. On the other hand, a constant decrease in *m*_s_ amplitudes is observed for comp. strains (−1% to −5%) as displayed in Fig. 12(a)/(b). In contrast, the Ru ion behaves differently and shows a slight increase in *m*_s_ under comp. strains. However, as tens. strain increases, its *m*_s_ value progressively decreases in both types of strains. Along with this, SP values against biax./hydro. strain are plotted in Fig. 12(a′)/(b′). Under biax. strain, SP gradually drops from 100% at ≤−3% to 79% at −4% and finally becomes 51% at −5%. Likewise, under hydro. strain, SP decreases significantly from 100% at ≤−3% to 48% at −4% and becomes very small at 2.3% at −5% (see Fig. 12(a′)/(b′)). This comparison highlights a major distinction between the two strain types: biax. strain retains a larger degree of SP compared to hydro. strain but SP declines under both situations beyond −4% because an electronic transformation from HM-to-metal is predicted at this strain level in both strain types.

**Fig. 13:**
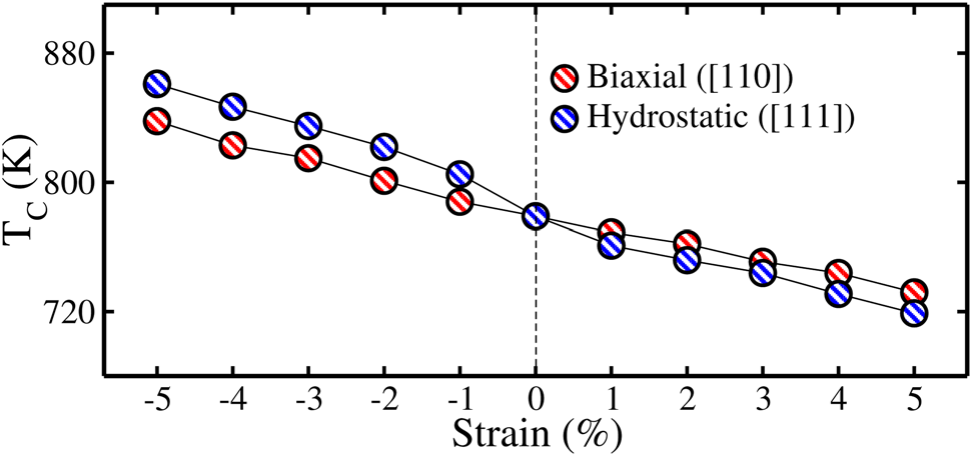
mBJ-GGA calculated Curie temperature (*T*_C_) under ±5% biaxial ([110]) and hydrostatic ([111]) strains in the CoFeRuSn quaternary Heusler alloy.

Furthermore, the significant strain-dependent behavior of the calculated *T*_C_ values for the CFRS motif under both strains is shown in Fig. 13. At −5% biax./hydro. comp. strain, the highest *T*_C_ of 838/861 K is recorded. Conversely, tens. strain reduces *T*_C_ consistently, with a minimal value of 719/732 K for biax./hydro. strain at +5%. The spin-filter effects of QHA CFRS owing to their robust HM nature with their high *T*_C_, supports the development of scalable devices that maintain performance across varying temp. Along with this, the large SP in QHA allows for efficient spin injection, which is vital for devices like spin-transfer torque magnetoresistive random-access memory.^76^ Further, CFRS’s high *T*_C_ enables strong magnetic ordering above ambient temp., making it ideal candidate for high-temp. spintronic applications such as magnetic sensors, spin filters, and non-volatile memory devices. Furthermore, strain-induced improvement of its TE characteristics, notably the improvement of *ZT* under comp. strain, improves the efficiency of high-temp. energy harvesting. Further, its electronic and thermal transport characteristics can be tuned using strain engineering, allowing it to behave consistently throughout a wide temp. range, making it a good choice for spintronic and TE applications under extreme environments.^47^

Moreover, Fig. 6S of the ESI† shows how the *ZT* value varies under hydro. strain, where comp. strain of −5%/−3%/−1% shows increasing *ZT* values, notably in *N*↓, reaching a highest value of 0.941 at −1% (see Fig. 6S(c) of the ESI).† Under tens. strain of +1%/+3%/+5%, *ZT* first declines, but subsequently increases and reaches a maximum value of 0.946 at +5% (see Fig. 6S(a′) of the ESI).† Likewise, Fig. 7S of the ESI† shows *ZT* values under biax. strain It is clear that comp. strain increases the *ZT* to a maximum value of 0.75 at −3% (see Fig. 7S(b) of the ESI),† whereas tens. strain increases the *ZT* value to 0.84 at +5% strain.

## 4 Conclusion

To summarize, the structural, electronic, magnetic, and thermoelectric traits of the unstrained and strained (biaxial ([110]) and hydrostatic ([111])) CoFeRuSn quaternary Heusler alloy are explored *via ab initio* calculations. Our results reveal that CoFeRuSn alloy is rigorously stable in the T1 configuration and thermodynamically stable with negative formation energies/high cohesive energies. A half-metallic ferromagnetic (HM FM) behavior is predicted in the unstrained system, which is defined by metallic conductivity in the spin-majority channel (*N*↑) and a semiconducting nature with an energy gap (*E*_g_) of 0.42 eV in the spin-minority channel (*N*↓). This HM nature is caused by Ru-4d orbitals around the Fermi level, with additional contributions from Co and Fe 3d states. The system retains strong magnetic ordering with a high Curie temperature (*T*_C_) of 779 K, suggesting substantial thermal stability. Furthermore, the magnetic characteristics are mostly regulated by the Co/Fe/Ru ions with magnetic moments of 1.59/3.13/0.53 *μ*_B_. The figure of merit (*ZT*) in an unstrained system is 0.99/0.93 at 150/300 K. Under hydrostatic (hydro.) and biaxial (biax.) strain, the *E*_g_ in the spin-minority channel (*E*^*N*↓^_g_) decreases under compressive (comp.) strain and leads to a semiconductor-to-metal transition at −4%, whereas tensile (tens.) strain improves the semiconducting state. Moreover, comp. strain also improves thermoelectric performance, with the spin-minority channel showing the maximum *ZT* value at +5% tens. strain. In addition, the Curie temperature (*T*_C_) attains a maximum value of 838/861 K at −5% biax./hydro. strain and subsequently declines with the application of increasing tensile strain. As CoFeRuSn exhibits a very high *T*_C_ along with a probable HM state it is an attractive material for spintronic applications in magnetic storage technologies such as hard disk drives and magnetic random-access memory.

## Data availability

The data used during the current study are available from the corresponding author on reasonable request.

## Author contributions

Farwa Rani: writing – original draft, investigations, formal analysis, data curation Bassem F. Felemban: validation, visualization, formal analysis. Hafiz Tauqeer Ali: writing, investigations, and formal analysis. S. Nazir: writing – review and editing, validation, supervision, project administration, conceptualization.

## Conflicts of interest

The authors declare no competing interests.

## Funding

This research was funded by Taif University, Taif, Saudi Arabia, Project No. (TU-DSPP-2024-133).

## Supplementary Material

RA-015-D5RA01305D-s001
