# Correction: Half metal-to-metal transition and superior transport response with a very high Curie-temperature in CoFeRuSn: strain regulations

**DOI:** 10.1039/d5ra90047f

**Published:** 2025-05-01

**Authors:** Farwa Rani, Bassem F. Felemban, Hafiz Tauqeer Ali, S. Nazir

**Affiliations:** a Department of Physics, University of Sargodha 40100 Sargodha Pakistan safdar.nazir@uos.edu.pk +92-334-9719060; b Department of Mechanical Engineering, College of Engineering, Taif University Kingdom of Saudi Arabia

## Abstract

Correction for ‘Half metal-to-metal transition and superior transport response with a very high Curie-temperature in CoFeRuSn: strain regulations’ by Farwa Rani *et al.*, *RSC Adv.*, 2025, **15**, 11511–11522, https://doi.org/10.1039/D5RA01305D.

The authors regret that the affiliation for the first author Farwa Rani (affiliation *a*) was incorrectly shown in the original manuscript. The corrected list of affiliations is as shown above.

The Royal Society of Chemistry apologises for these errors and any consequent inconvenience to authors and readers.

