# Supplementary material for: Half metal-to-metal transition and superior transport response with a very high Curie-temperature in CoFeRuSn: strain regulations
Source: RSC Adv. 2025 Apr 11;15(15):11511–22. doi: 10.1039/d5ra01305d (PMC11987858; doi:10.1039/d5ra01305d)
Supplement: RA-015-D5RA01305D-s001 [file RA-015-D5RA01305D-s001.pdf]

Supplementary Information (SI) for RSC Advances.
This journal is © The Royal Society of Chemistry 2025

Supporting Information

Half Metal-to-Metal Transition and Superior Transport Response Holding a Very High Curie-Temperature in CoFeRuSn: Strain Regulations

Farwa Rani[1], Bassem F. Felemban[2], Hafiz Tauqeer Ali[2], and S. Nazir[1*]
[1]*Department of Physics, University of Sargodha, 40100 Sargodha, Pakistan and*
[2]*Department of Mechanical Engineering, College of Engineering, Taif University, Kingdom of Saudi Arabia*

* Electronic address: safdar.nazir@uos.edu.pk, Tel: +92-334-971-9060

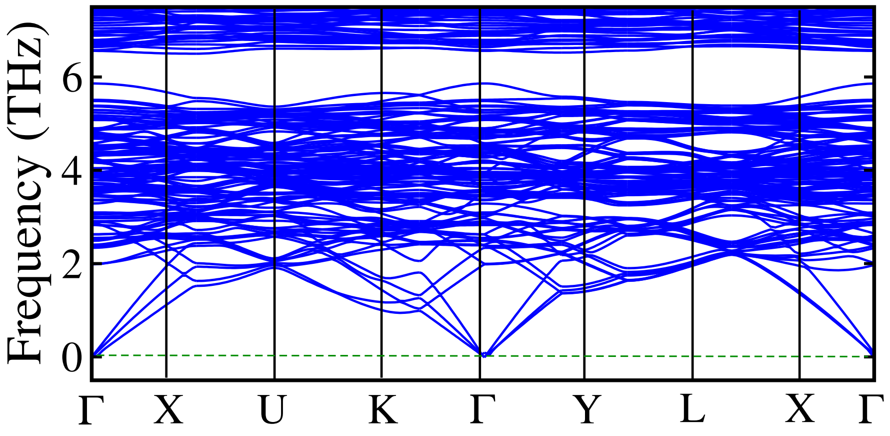

FIG. 1S: Calculated Phonon bands of the CoFeRuSn quaternary Heusler alloy.

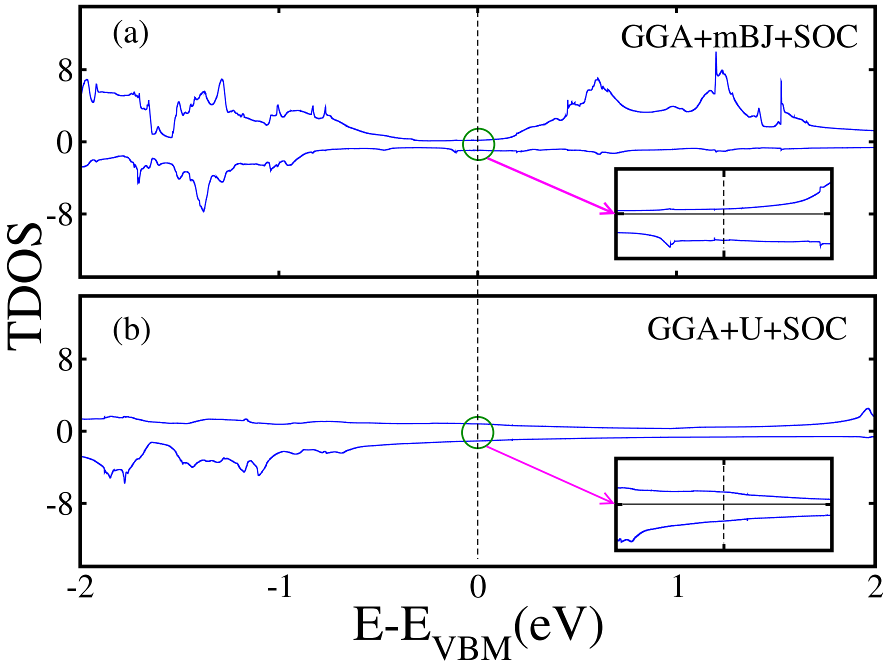

FIG. 2S: Computed (a/b) GGA+mBJ+SOC/GGA+U+SOC spin non-degenerated total density of states (TDOS) in states/eV in a stable T1 configuration for the unstrained CoFeRuSn quaternary Heusler alloy.

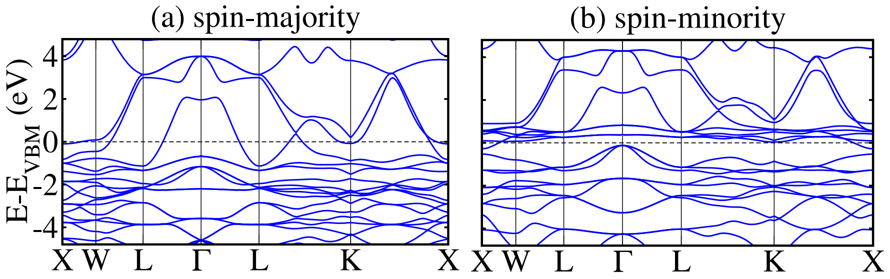

FIG. 3S: GGA calculated non-degenerated band structures for the spin-majority/spin-minority channel in (a/b) of the CoFeRuSn quaternary Heusler alloy in a stable T1 configuration.

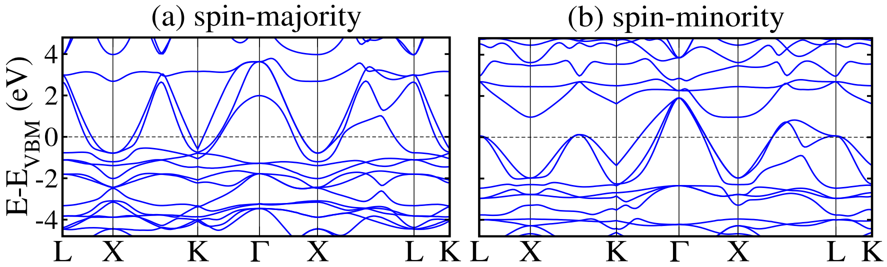

FIG. 4S: GGA+U calculated non-degenerated band structures for the spin-majority/spin-minority channel in (a/b) of the CoFeRuSn quaternary Heusler alloy in a stable T1 configuration.

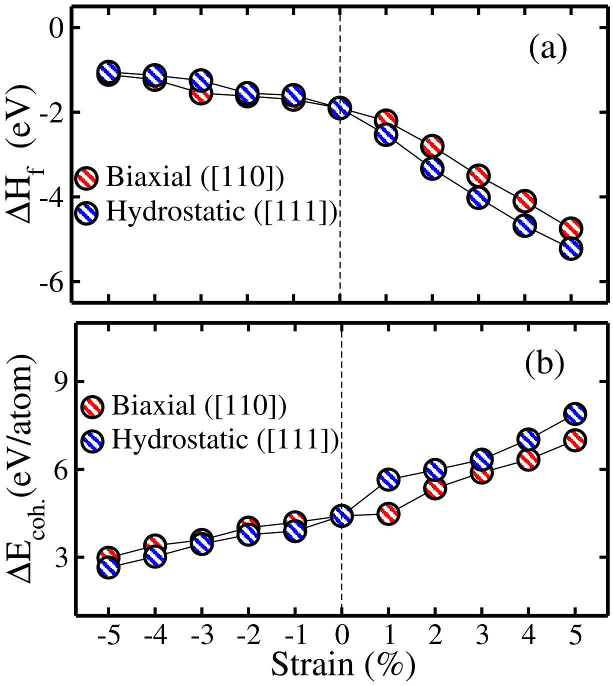

FIG. 5S: mBJ-GGA computed (a) enthalpies of formation (ΔH_f) and (b) cohesive energy ($\Delta E_{coh.}$) as a function of ± 5% biaxial ([110])/hydrostatic ([111]) strain in the CoFeRuSn quaternary Heusler alloy.

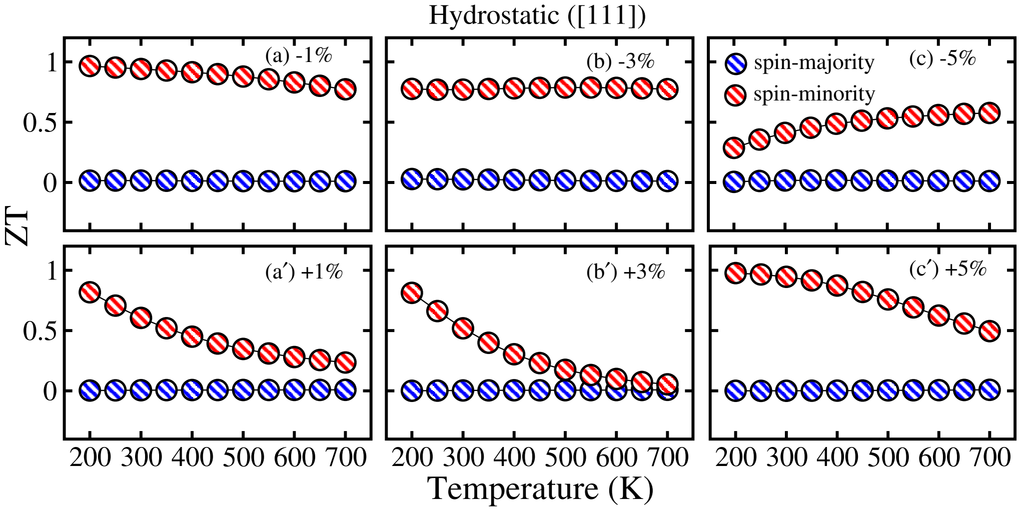

FIG. 6S: Computed figure of merit (ZT) as a function of temperature for (a/a′) −1%/+1%, (b/b′) −3%/+3% and (c/c′) −5%/+5% for hydrostatic ([111]) compressive/tensile strain in the CoFeRuSn quaternary Heusler alloy.

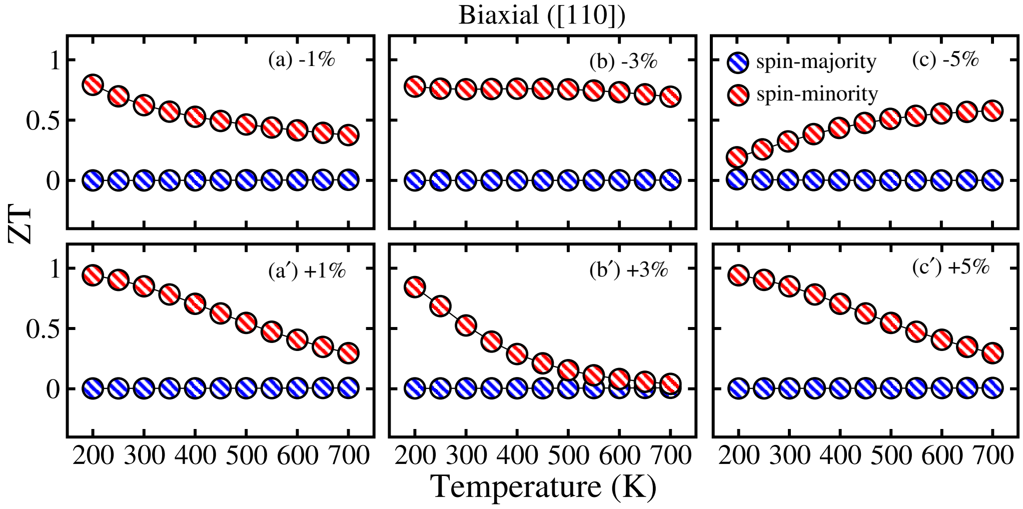

FIG. 7S: Computed figure of merit (ZT) as a function of temperature for (a/a′) −1%/+1%, (b/b′) −3%/+3% and (c/c′) −5%/+5% for biaxial ([110]) compressive/tensile strain in the CoFeRuSn quaternary Heusler alloy.
